# Assessment in Sport and Exercise Psychology: Considerations and Recommendations for Translation and Validation of Questionnaires

**DOI:** 10.3389/fpsyg.2022.806176

**Published:** 2022-03-03

**Authors:** Luis Cid, Diogo Monteiro, Diogo Santos Teixeira, Anastasiia Evmenenko, Ana Andrade, Teresa Bento, Anabela Vitorino, Nuno Couto, Filipe Rodrigues

**Affiliations:** ^1^Sport Sciences School of Rio Maior, Polytechnic Institute of Santarém (ESDRM-IPSantarém), Rio Maior, Portugal; ^2^Research Centre in Sports Sciences, Health Sciences and Human Development (CIDESD), Vila Real, Portugal; ^3^Quality of Life Research Center (CIEQV), Santarém, Portugal; ^4^ESECS, Polytechnic of Leiria, Leiria, Portugal; ^5^Faculty of Physical Education and Sport, Lusófona University (ULHT), Lisbon, Portugal; ^6^Research Center in Sport, Physical Education, and Exercise and Health (CIDEFES), Lisbon, Portugal

**Keywords:** psychological assessment, sport, exercise, physical activity, physical education, questionnaire validation, factor analysis

## Abstract

Translating and validating measurement instruments in sport and exercise psychology is not an easy task. Rather, it is a task that requires effort and time, for the process is not limited to a simple translation to translate words from one language to another, just in order to make valid and reliable measure. All researchers should be aware that the only proper way is to adopt rigorous and robust methodologies to conduct the process from the preliminary stage of translation to reaching the validation stage of the psychological variable. Only so is it possible to avoid creating fragile and inadequate psychological assessment instruments that can jeopardize the entire investigation to be held with its use. Thus, the main objective of this work is to promote reflection and discussion on the subject by presenting some considerations and recommendations about translation and validation of questionnaires for psychological assessment applied to sport and exercise domain.

## Introduction

Psychological assessment can be defined as a scientific and professional activity that consists of collecting, integrating and analyzing data about a specific individual (Anastasi and Urbina, 1997). However, one of the main problems we face is the lack of instruments properly adapted or validated to a specific population, in the most varied application contexts (Gonçalves et al., 2006). In the authors’ opinion, this issue originates, in most cases, abusive uses of measuring instruments in psychology (e.g., use of criteria validated in other countries or lack of support of a conceptual nature).

In the context of sport and exercise, the paradigm is not very different. According to the directory of psychological tests for sport and exercise (see: Ostrow, 1996), the number of tests applied to sport and exercise was very small by the end of the twentieth century. There have been about 300 tests that were used in this context, although only about a half of them were developed or validated specifically for sport. Despite the proliferation of their use since the 1st World Congress of Sport Psychology (held in Rome in 1965), the number is still very limited (Ostrow, 2001). However, with the turn of the century, especially in the last decade, the situation has changed significantly.

In the specific case of Portugal, where psychological assessment instruments developed specifically for sport and exercise are not abundant, researchers have two options for action (Fonseca and Brito, 2005): (1) to develop new instruments; and (2) make adaptations of the instruments that already exist in other languages to the Portuguese language. In the authors’ opinion, the second option is the most favorable, since the results between cultures can be compared, the excessive creation of instruments measuring the same constructs is avoided, and as a result, the measurements become more robust.

Assuming in the first place that a psychological test (e.g., questionnaires) is an objective and standardized measure of a sample of behavior, developed and used to determine and analyze personal differences or the reactions of the same person on different occasions (Anastasi and Urbina, 1997), it can be easily inferred that the expression “objective and standardized measure” refers to the psychometric qualities and to the uniformity of the measure in terms of its application, correction and interpretation.

As a matter of fact, a good psychological test should meet three main criteria (Allworth and Passmore, 2008): (a) it should represent an accurate measure of the psychological attribute (i.e., have both validity and reliability); (b) it should help differentiate individuals regarding the psychological attribute in question (sensitivity); and (c) it should be a good indicator of future behavior (predictive value). Also, it should be noted that it is through the items of a questionnaire (observable variables) that inferences about behavior are made and latent psychological attributes are measured (non-observable variables), therefore, it is essential that the instruments are objective, accurate and assess exactly what is necessary to be measured (Fachel and Camey, 2003).

In short, a well-developed (or translated), valid and reliable psychological test is one that undergoes a rigorous process during its development (Allworth and Passmore, 2008). However, this is not what happens in most cases carried out in the field of sport and exercise psychology. The issue of instrument validation in this context is still largely neglected and many instruments are subject to questionable adaptation processes, which either compromise the evaluation results. For this reason, malpractice should not happen, and the procedures should be more rigorous and robust, so that there are no doubts about the psychometric quality of the translated versions (Fonseca and Brito, 2005). In this sense, the methodological approach should be strong, allowing for a clear interpretation that can be drawn from the results and for the identification of the strengths and weaknesses. Thus, the main aim of this work is to promote reflection on the topic, presenting some considerations and recommendations for the translation and validation of psychological assessment questionnaires applied in the context of sport and exercise.

## Translating of a Questionnaire

Translating assessment psychological instruments, in order for them to be used in other cultures involves more than simply translating a text into another language (Vijver and Hambleton, 1996). For the translation of any questionnaire from its original language (e.g., English) to Portuguese, rigorous methodological procedures must be adopted, which establish the relevance of the instrument taking into account aspects related to factors and concepts specific to a given culture *(emic concepts)*, as well as aspects related to factors and concepts that are universal to all cultures *(etic concepts)* (Duda and Hayashi, 1998; Banville et al., 2000; Geisinger and McCormick, 2012).

Aiming at filling the gap in the literature, Vallerand (1989) developed a specific methodology for the cross-cultural adaptation of psychological questionnaires, systematized in 7 steps: (1) Preparation of a preliminary version, using the translation/back translation technique (see: Brislin, 1970). The author suggests the use of two translators and two backward translators; (2) Evaluation of the preliminary version and preparation of an experimental version to verify that the retroverted version accurately reflects the original version. The author suggests an evaluation panel of 3 to 5 people (which should include the two translators and researchers); (3) Pre-test of the experimental version, applied in a sample of the target population. The number of people is not important as no statistical technique will be applied; (4) Evaluation of concurrent and content validity (the latter by the previously mentioned evaluation panel). The author suggests 20 to 30 bilingual individuals from the target population, since if there is no concurrent instrument already validated, both versions of the questionnaire (original and translated) should be applied simultaneously; (5) Assessment of factor reliability through the analysis of temporal stability (test-retest) with an interval of 4 weeks, and through the analysis of internal consistency (Cronbach’s alpha). The author does not refer to the number of individuals to be involved in this phase, but indicates values for the correlations (*r* > 0.60) and alphas (α > 0.70) for good reliability; (6) Construct validity assessment to verify whether in the new culture the translated instrument does measure the theoretical construct it should supposedly measure. The author only suggests that the structure of the questionnaire be verified through factor analysis; and (7) Establishment of rules for application, correction and interpretation of results, so that the subject can be compared with an appropriate reference group. The author suggests for a larger number of subjects and the presentation of simple statistical results (mean, standard deviation and percentiles).

According to Banville et al. (2000), the methodology proposed by Vallerand (1989) represents an effort that should be made to take into account the cultural peculiarities in which the instrument will be used. However, despite being the most used, this methodology is not the only one, since, according to the authors, in several works the translation/backward translation technique is not used. In fact, some disadvantages of this technique can be pointed out, namely: (a) it is not at all acceptable to hand this task over to simple translators, since at the translation stage it is essential to have a strong knowledge of the psychological attributes and application context; b) the retroverted version rarely replicates the questionnaire items exactly as they were in their original form, so addition time should be usually allocated to detect and confront these differences.

Perhaps, these were the reasons that led Fonseca and Brito (2005) to suggest the constitution of bilingual juries (panels of experts in different areas of knowledge) to evaluate the initially translated version, thus replacing the translation/backward translation technique. This method, called *the committee approach* (see: Brislin, 1980), consists of the assessment of the instrument by a group of bilingual people who have in-depth knowledge of the theoretical constructs measured by the questionnaire, understand the specifics of the context of application and who are familiar with the basic principles of psychological assessment (Geisinger and McCormick, 2012). In the opinion of Fonseca and Brito (2005), this method is not only not new, but it had also been recommended by several authors, since it can represent an improvement in the quality of the assessment of the semantic aspects of the instruments. Furthermore, according to Geisinger and McCormick (2012), this method has the advantage of allowing committee members to more easily detect possible errors inherent to the translation process, through cooperation within each one’s specialization.

However, regardless of the method used, it is vital to establish the meaning (semantic value) of the items in the original questionnaire, so that they are kept in the translated version. Thus, the following recommendations should be taken into account (see: Vijver and Hambleton, 1996): (1) literal translations are not essential and should be avoided; (2) priority should be given to the semantic aspect of the items in detriment to the literal translation; (3) it is extremely important, useful and necessary to know and understand the concepts/theoretical models underlying the assessment instruments; and (4) it is essential to take into account the application context and the target population.

In sum, in line with the aforementioned recommendations, our methodological suggestion for the translation of questionnaires in the field of sport and exercise psychology encompasses the following steps:

(1) Authorization: asking the author of the original version for their authorization to carry out the translation is a recommended ethical rule, and has some advantages: (a) knowing if any other translation has already been carried out; (b) obtaining the author’s collaboration in carrying out the study; (c) receiving additional information about the questionnaire; and (d) the author learns that his questionnaire will be available in another language;
(2) Preliminary Translation: this step must be carried out by researchers with the help of translators with in-depth knowledge of the original language and their mother tongue (e.g., higher education and specific knowledge of translation techniques). It is advisable to use help of 3 translators (two at least). This first version of the translation is the responsibility of the researchers, after receiving and analyzing the translators’ suggestions;
(3) First Evaluation: the analysis/evaluation of the initial version should be carried out by an evaluation committee (committee approach), composed of 4 or 5 experts from different areas of knowledge (e.g., one with a Degree in Languages; one Psychologist, one or two Sport Psychologists, one with a Degree in Sport Science). The members of the evaluation committee should individually present comments and/or suggestions for change. The second version of the translation is the responsibility of the researchers, after collecting and analyzing the suggestions of the members of the evaluation committee;
(4) Second Evaluation: the second version of the translation is resubmitted for analysis/evaluation by another independent evaluation committee of the first one, equally composed of four or five specialists (e.g., one Psychologist, two or three Sport Psychologists, one Graduated in Sport Sciences). At this stage, the members of the committee present their comments and/or suggestions for changes. The researchers, after collecting the opinions of the members of the committee, will organize and moderate a group meeting, in which the discrepancies of opinions existing among the members of the committee on each of the items of the questionnaire should be analyzed and discussed. This phase only ends when there is agreement between the experts, and the opinion of all members of the jury has been unanimous in regards to the final content. This results in the third version of the translation;
(5) Pilot Study: elaboration of the first layout of the questionnaire (including instructions) and its application to 50 subjects from the target population (number suggested by: Hill and Hill, 2005), for analysis and determination of difficulties in understanding and interpretation. Participants should be invited and encouraged to indicate directly in the questionnaire the words or expressions they do not understand, as well as to make comments and/or suggestions for changes (including the layout and instructions). The researchers should review all comments and make appropriate changes (if applicable). This step results in the fourth version of the translation;
(6) Final Review: revision of the language – syntax aspects: spelling, grammar, punctuation and phrasing (e.g., carried out by two graduates in the language of the questionnaire). This results in the final version to be validated.

## Assessment of the Reliability and Validity of a Questionnaire

Psychological tests should respect psychometric criteria typical of most of these measures, which relate to two major types of metric properties: reliability and validity. Therefore, when talking about reliability, it is necessary to take two main aspects into account: the stability and consistency of the results of the observable variables (Hill and Hill, 2005). According to these authors, no psychological measure is perfect, so there is always an inevitable error margin associated: reliability = 1 – measurement error variance/variance of observed values. This formula derives from the classical error theory, which suggests that the result of any measurement instrument is determined by the true value of the psychological attribute (latent variable), plus the associated measurement error. However, reliability also refers to the degree of stability by which the result of a subject, when answering the questionnaire, remains relatively consistent after repeated application of the same. The perfect instrument is the one that always produces the same result for the same subject (Schutz and Park, 2004), although this is very unlikely to happen in psychology due to the errors associated with the measurement. In any case, standardization of the instrument always results in a reduction of this error, and for this reason, it is always necessary to analyze two types of reliability in any instrument (Nideffer and Sagal, 2001; Hill and Hill, 2005; Buckworth et al., 2013): evaluation of temporal reliability (measurement stability) and internal reliability assessment (internal consistency).

In the case of temporal reliability, the assessment must be carried out through a test-retest analysis of the results of the items and factors of the questionnaire (Pearson correlation coefficient – *Pearson’s r*), based on its application to the same subject at two different times (always in conditions of similar application). This type of analysis allows us to assume that the questions raised are clear and refer to relatively stable aspects over time (Vallerand, 1989; Nideffer and Sagal, 2001; Fachel and Camey, 2003; Schutz and Park, 2004; Hill and Hill, 2005; Banville et al., 2000). According to Noar (2003), although in psychology it is very unlikely to obtain very high correlations, the higher the correlation coefficient, the greater the temporal reliability. The values reported in literature indicate 0.70 as an acceptable minimum (Nideffer and Sagal, 2001; Allworth and Passmore, 2008), although Vallerand (1989) indicates a value of 0.60 as satisfactory. Regarding the number of subjects to be used, taking into account the statistical technique involved, we suggest at least 30 (see: Hill and Hill, 2005).

Regarding the definition of the time interval between the two applications, two problems arise (Fachel and Camey, 2003; Moreira, 2004): (1) shorter time intervals that increase the risk of memory interference and of the subjects still remembering the responses given in the first application, so there may be a tendency to respond equally; and (2) longer time intervals increase the risk of cognitive, affective or behavioral changes in the subjects, so there may be an effective change in the results. Therefore, intervals shorter than 1 week or longer than 1 month should be avoided, respectively, because they might imply a considerable memory factor, or because they may pose problems regarding possible changes (Moreira, 2004). Consequently, an interval between two (Nideffer and Sagal, 2001) to four (Vallerand, 1989) weeks can be considered the most adequate.

When analyzing internal reliability, the assessment should be performed using Cronbach’s alpha (α), which analyzes the extent to which the items contribute to measuring the same factor. If the value of alpha is 1, we are faced with perfect internal reliability. However, very high *alpha* values may indicate a certain redundancy between questionnaire items (Vallerand, 1989). In any case, the researchers can adopt as reference the following ranges of values (see: Hill and Hill, 2005): α < 0.60 – unacceptable; α = 0.60–069 – weak; α = 0.70–0.79 – reasonable; α = 0.80–0.89 – good; α > 0.89 – excellent. However, in confirmatory factorial analysis, the assessment should be performed using composite reliability since is a measure of internal consistency reliability, which, in contrast to Cronbach’s alpha, does not assume equally weighted indicator loadings. Composite reliability should be above 0.60 in exploratory research, and above 0.70 as a general guideline, but not above 0.95 (Hair et al., 2019). Raykov’s formula must be used to calculate composite reliability (see Raykov, 1997).

On the other hand, when talking about validity, we are not referring to its results (as it happens in reliability), but rather to the relationship between the results and something that underlies them: an inference or action (Moreira, 2004). According to this author, the essence of the validity of a test lies in the inferences that we can make through its results (meaning) and in the consequences of its use to guide actions (utility). Therefore, to check the validity of a questionnaire the following question needs to be answered: does the test measure what it is supposed to measure (Fachel and Camey, 2003)? In our opinion, it is essential that this issue be also properly framed in terms of the target population and the specific context of application of the instrument (e.g., sport, exercise or other physical activity domain).

In scientific literature some discrepancies between authors about the names and concepts of validity can be found. However, according to the American Psychological Association (see: American Psychological Association [APA], 1985), validity can be grouped into three categories: (a) content-related validity, which is the “theoretical” examination of the test content that determines whether its items are appropriate and relevant (i.e., content validity and face validity); (b) criterion-related validity, which is the examination of the “quality” of the test as a present or future predictor of another variable (i.e., concurrent validity and predictive validity); and (c) construct-related validity, which is the examination of the theoretical “concept” that underlies the test (i.e., convergent validity, discriminant validity and factorial validity).

These three types of conception of validity have originated some blurring of the validity status as a whole, although there is a tendency to blur the boundaries between the different types of validity and to emphasize their unitary character (see Schutz and Park, 2004). The progressive affirmation of this unitary character has been accentuated both at the conceptual and at the methodological level. It is important to highlight that the progressive unification has taken place around the construct validity (Moreira, 2004), which is the noble validity of any measure, since it is what guarantees that the test measures the psychological attribute in question.

According to Buckworth et al. (2013), psychometry is based on the assumption that unobservable psychological variables can be measured indirectly by inference. However, this inference should be composed by a logical pattern of associations between the subject’s perception of their own experiences, and the behaviors, as well as the social context in which they occur. Therefore, psychometry should be based on a process of construct validity, through which this pattern of associations is established. Therefore, it is advisable for researchers to assess different types of validity of their instruments, namely: content (to ensure that the indicators are representative of the construct), factorial (to determine the structure of the construct), convergent (to verify that the indicators are related to the construct) and discriminant (to verify that the construct is independent).

Although we will approach factorial validity in more detail below, we would like to highlight the importance of convergent and discriminant validity, especially when we use confirmatory factor analysis. According Hair et al. (2019), convergent validity measures the extent to which the indicators of a construct converge, thereby explaining the variance of the item, and it is assessed by evaluating the average variance extracted (AVE) across all indicators associated with a particular construct, considering values of ≤ 0.50. The AVE is the average (mean) of the squared loadings of all indicators associated with a particular construct. Discriminant validity evaluates the extent to which a construct is distinct from other constructs. The underlying principle of discriminant validity is to assess how uniquely the indicators of a construct represent that construct (the shared variance within that construct) versus how much that construct is correlated with all other constructs in the model (shared variance between constructs). Using the concept of AVE mentioned above, discriminant validity is present when AVE for each construct exceeded the squared correlation between that construct and any other.

## Exploratory Factor Analysis (EFA)

**FIGURE 1:**
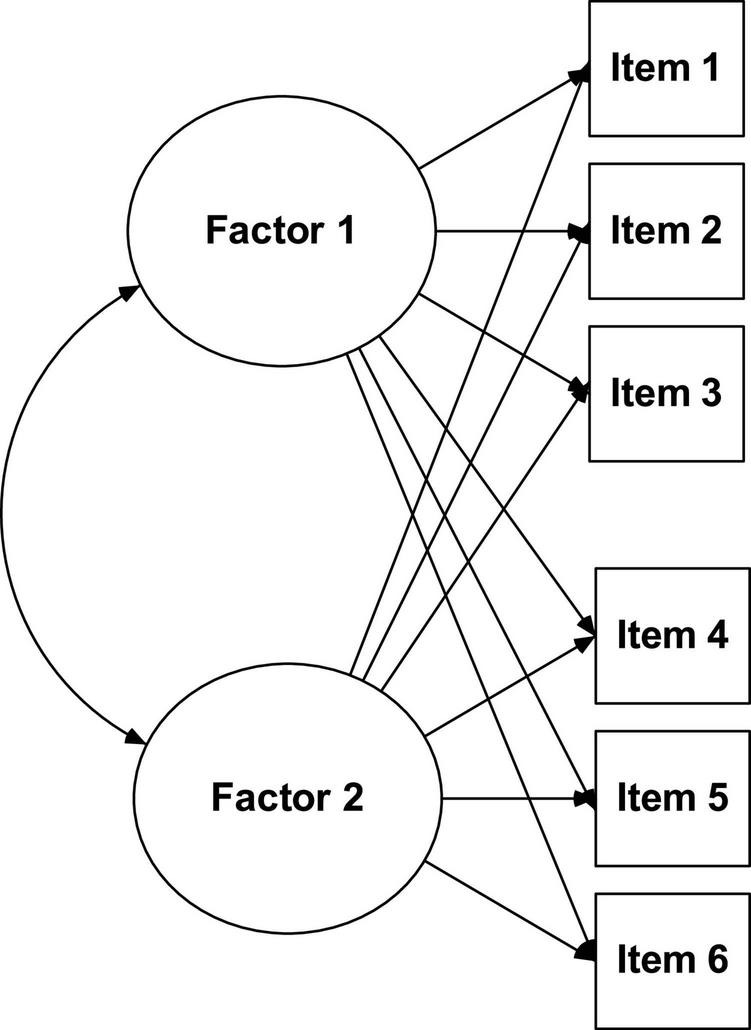
Exploratory factor analysis.

Exploratory factor analysis (see Figure 1) makes it possible for a large number of variables to be reduced to factors by exploring the correlations between the observable variables (items), allows its grouping in dimensions (latent variables), estimating the number of factors that are necessary to explain the variance of the items, as well as the structural relationships that link them to each other (Moreira, 2004). This type of analysis is widely used when researchers *a priori* do not have any assumptions about the nature of the factor structure of their data. However, it is also quite common at a preliminary stage of instrument validation, even when there are indications about the factors provided by a theoretical model.

According to various authors (Hill and Hill, 2005; Worthington and Whittaker, 2006), there must be a high correlation between the variables for EFA to be useful in the estimation of common factors. The measure of adequacy of the sampling of *Kaiser-Meyer-Olkin* (KMO test) and the *Bartlett* sphericity test (Bartlett test) are the most used ones to assess the quality of correlations in order to proceed (or not) with the EFA. It is recommended that the KMO test value be greater than 0.6 and the Bartlett test be associated with a significant *p*-value.

The most used methods for factor extraction are: common factor analysis (*FA*) and principal components analysis (*PCA*) (Henson and Roberts, 2006; Kahn, 2006; Worthington and Whittaker, 2006; Hair et al., 2019; Tabachnick and Fidell, 2019). Theoretically speaking, the main difference lies in the fact that PCA has as its main objective the analysis of the total variance (common, specific and error), while the FA has as its main objective analyzing only the common variance in a set of variables to understand or explain the correlations between them (Hill and Hill, 2005). In practical terms, both methods produce very similar results. However, they should be used depending on their characteristics (Hair et al., 2019): PCA to reduce the data to a minimum number of factors; FA (e.g., principal axis factoring) to identify latent constructs in a set of variables, when there is already some defined theoretical specification.

Initial EFA results usually do not produce an easily interpretable structure (Kahn, 2006), so factor rotation is the most important tool (Hair et al., 2019), as it produces a clearer and more objective factorial solution, which maximizes the factorial weights of the items (Brown, 2015). However, a question arises: *which rotation method to use (oblique or orthogonal)*?

According to several authors (Preacher and MacCallum, 2003; Henson and Roberts, 2006; Kahn, 2006; Worthington and Whittaker, 2006; Brown, 2015; Hair et al., 2019; Tabachnick and Fidell, 2019), the decision must be taken depending on the expected correlation between the factors. If in theoretical terms it is plausible that the factors are not correlated, then we should use an orthogonal (orthogonal) rotation. But if the factors are theoretically expected to correlate with each other, then the best option is an oblique rotation. In the first case, the most used rotation is *Varimax*, in the second case the best option is *Promax*. However, although the results are slightly different depending on the type of rotation, these differences are usually not drastic (Kahn, 2006).

According to Preacher and MacCallum (2003) the mechanized use of the Varimax orthogonal rotation should be avoided, since its use is rarely sustained, as the factors are almost never independent. Furthermore, the *Promax* oblique rotation is almost always the best choice, because the analysis starts with an orthogonal rotation and ends with an oblique solution, i.e., if the factors are independent, the rotation remains orthogonal, but if they are correlated, the rotation will be oblique (Kahn, 2006). In our opinion, most researchers use orthogonal rotations *(Varimax)* because they only produce a matrix of results *(rotated matrix)*, which facilitates the interpretation of the solution found. On the contrary, oblique solutions produce two result matrices: the configuration matrix (*pattern matrix* which indicates the unique contribution of each item to the factor) and the *structure matrix*, which in addition to indicating the contribution of each item for the factor, also takes into account the relationship between the factors (Hair et al., 2019). Although it is not consensual which of the matrices should be used, it is the configuration matrix that is most frequently interpreted and reported in applied research (Brown, 2015). The reason for this is that the structure matrix results tend to be overestimated due to the increased correlation between factors.

After choosing the analysis methods to be used, another fundamental question arises: what are the criteria for determining factors and retaining or eliminating items? The answer is not easy, as there are numerous guidelines and recommendations available in literature (Preacher and MacCallum, 2003; Henson and Roberts, 2006; Kahn, 2006; Worthington and Whittaker, 2006; Blunch, 2013; Brown, 2015; Hair et al., 2019; Tabachnick and Fidell, 2019). Therefore, at the time of decision, we suggest that the following combination of criteria be taken into account:

(1) *Kaiser’s criterion*: measure of explained variance defined in the same metric as the items. The decision rule tells us to retain factors with eigenvalue equal to or greater than 1 (*eigenvalue*: EV ≥ 1.0). Low unit values reflect instability in the factor. In addition, one should also analyze the *“elbow” graph (scree plot)* and observe the number of factors above the “elbow bend,” despite its subjective nature (Kahn, 2006);
(2) *Communalities:* proportion of the variance of each item that is explained by the set of extracted factors. Hair et al. (2019) advise researchers to analyze them from an orientation perspective, checking whether the items reach acceptable levels or not. Values above 0.50 indicate that a good part of the variance in the results of each item is explained by the factorial solution. However, the hypothesis of elimination of items should only be considered with values below 0.40 (Worthington and Whittaker, 2006). In any case, the value of commonalities should only function as a guide for decision-making and not as the main criterion;
(3) *Factor loading*: correlation between the item and the factor. Normally, factor weights are considered significant when the value is equal to or greater than 0.5 (FL ≥ 0.50). Some authors assume that the value 0.30 is relevant and considered as a minimum to be interpreted (Kahn, 2006; Worthington and Whittaker, 2006; Hair et al., 2019; Tabachnick and Fidell, 2019). Factor weights above 0.70 are considered indicative of a very well-defined structure, as the factor explains at least 50% of the item’s variance (factorial weight squared) (Hair et al., 2019; Tabachnick and Fidell, 2019). Alternatively, we can consider the values as a function of sample size (Hair et al., 2019): 0.30 (*n* > 350); 0.35 (*n* > 250); 0.40 (*n* > 200); 0.45 (*n* > 150); 0.50 (*n* > 120); 0.55 (*n* > 100); 0.60 (*n* > 85); 0.65 (*n* > 70); 0.70 (*n* > 60); 0.75 (*n* > 50);
(4) *Cross-loadings factorial weights.* Inexistence of items with relevant factor weights (above 0.30) in more than one factor. If this happens and if the difference between them is not equal to or greater than 0.15, we should consider eliminating the item (Worthington and Whittaker, 2006);
(5) *Percentage of variance explained by the factors*. Although there is no absolute rule on this issue, the aim is that factor extraction ensures a high amount of item variance. If this happens, it means that most of the variance of the observed variables is explained by latent factors and not by other aspects that are not known. The percentage of variance explained by the retained factors must be at least 40% (Blunch, 2013). Factor solutions that explain 60% of the data variance are considered very satisfactory (Hair et al., 2019);
(6) *Internal reliability*. The factor’s internal consistency must be equal to or greater than 0.70 (*Cronbach’s alpha:* α ≥ 0.70), although this value may decrease to 0.60 in the case of EFA (Hair et al., 2019). For guidance purposes, we suggest using the above-mentioned values (see: Hill and Hill, 2005). We must also analyze two additional aspects: the value of internal consistency in case of elimination of an item, it being necessary that the alpha value does not increase if this happens; and item-total correlations (correlations between the item and the total factor value), with values above 0.50 being advisable (Hair et al., 2019);
(7) *Retention of factors with at least three items.* This rule is paramount for reasons of model estimation at a further stage of instrument validation (Blunch, 2013; Brown, 2015; Kline, 2016). Good practices dictate a minimum number of three items per factor (preferably four) (Hair et al., 2019). According to these authors, having many items per factor is not necessarily the best option either, as it can bring about such problems as difficulties in producing a true one-dimensionality of the factor.

Some of these suggestions and/or recommendations have been taken into account by several authors in Portugal, in the validation of questionnaires (using EFA), in the area of sport and exercise psychology (Fonseca and Brito, 2005; Pires et al., 2010; Cid et al., 2012a,b).

Finally, we cannot fail to mention the number of subjects needed to perform the EFA. The 10:1 ratio (number of subjects for each item in the questionnaire) is the recommended number and is the one that generates some, yet not full consensus in the literature (Hill and Hill, 2005; Henson and Roberts, 2006; Kahn, 2006; Worthington and Whittaker, 2006; Hair et al., 2019). However, there are also authors who mention minimum ratios of 5:1 (Henson and Roberts, 2006; Hair et al., 2019) and others that mention absolute values: 50 very poor; 100 poor; 200 acceptable; 300 good (Tabachnick and Fidell, 2019).

## Confirmatory Factor Analysis (CFA)

**FIGURE 2:**
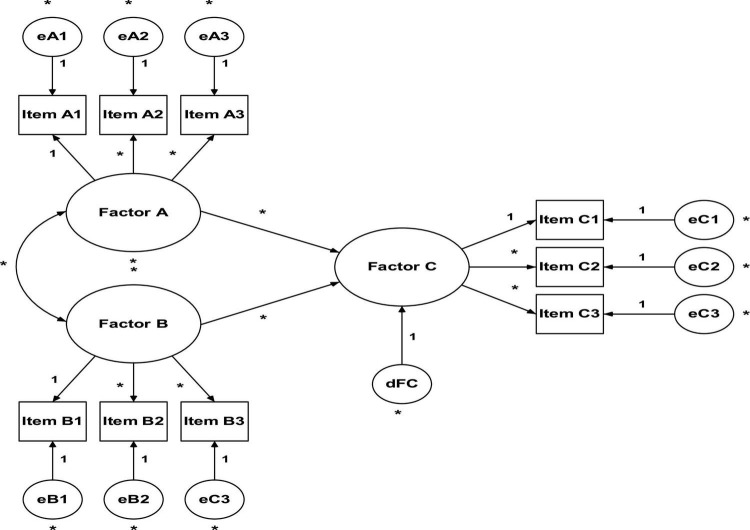
Structural equation modeling.

Structural Equation Modeling (SEM) can be seen as a fusion between two approaches to model evaluation: regression analysis and factor analysis. While regression analysis (also known as path analysis) is concerned with the causal relationships between variables, factor analysis is concerned with finding a set of factors that explain the common variance between a set of items (Biddle et al., 2001). In other words, SEM (see Figure 2) is a multivariate technique that allows the researcher to simultaneously examine the relationships between the latent constructs and the respective measurement variables, as well as between the various constructs of the model (Hair et al., 2019). Therefore, SEM has become the most used tool to explain theoretical models in social and human sciences, and its application is common during the development/translation process of questionnaires for construct validation after EFA (Worthington and Whittaker, 2006). According to Kahn (2006), researchers normally use the EFA to explore the correlations between the variables and identify possible factors that explain their variance, and the AFC to confirm whether the model structure fits the data well or not. Unlike what happens in EFA, where there are supposedly no *a priori* hypotheses about the number of factors and their relationship with the items, in AFC the model (number of factors, corresponding items and measurement errors) is defined and specified at the onset (Buckworth et al., 2013; Kline, 2016).

**FIGURE 3:**
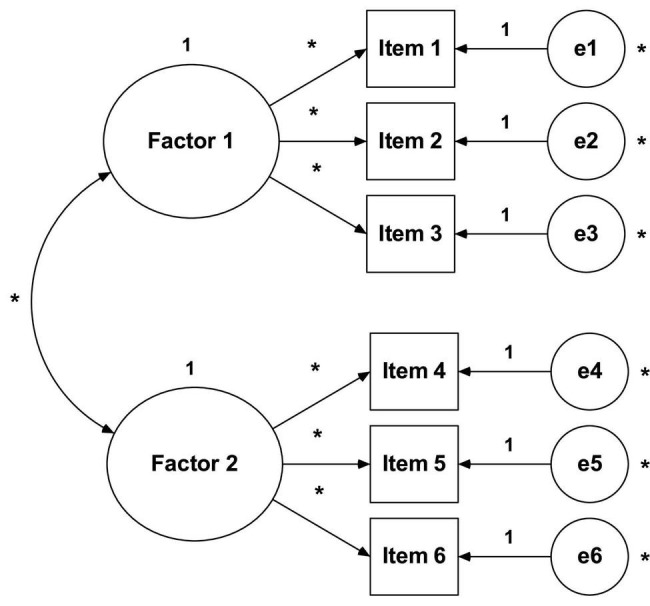
Confirmatory factor analysis.

In essence, both EFA and AFC aim to reproduce the observed relationship between the group of items and latent variables. However, the fundamental difference lies in the number and nature of the specifications/restrictions carried out *a priori*. CFA (see Figure 3) requires strong empirical or conceptual foundations to guide the specifications to be estimated in the model (Buckworth et al., 2013; Brown, 2015; Byrne, 2016; Kline, 2016; Hair et al., 2019).

The statistical theory underlying this type of analysis requires relatively large samples to perform the CFA (Kahn, 2006), a ratio of 10:1 being advised (number of individuals for each parameter to be estimated in the model and not per questionnaire item such as suggested for EFA) (Biddle et al., 2001; Worthington and Whittaker, 2006; Kline, 2016). Some authors use absolute minimum numbers: *n* ≥ 200 (Barrett, 2007) or 100 < *n* < 150 (Markland, 2007; Hair et al., 2019). Therefore, the best option is to take into account the complexity of the model (number of parameters to be estimated), and the following recommendations: minimum ratio of 5:1 (Bentler and Chou, 1987; Worthington and Whittaker, 2006); advised ratio of 10:1 (Bentler and Chou, 1987; Biddle et al., 2001; Worthington and Whittaker, 2006; Kline, 2016). To minimize the problem of non-normal data distribution using a ratio of 15:1 or large samples (*n* > 400) is advised (Hair et al., 2019).

The fundamental issue in model specification (definition of the free parameters to be estimated or fixed) is related to its identification, that is, the correspondence between the information to be estimated (free parameters), and the information from which it will be estimated (observed variances and covariances) (Hoyle, 1995). Therefore, the two requirements necessary for the model to be identified, that is, for the parameters to be estimable and the model to be analyzed, are (Chou and Bentler, 1995; MacCallum, 1995; Byrne, 2016; Kline, 2016; Hair et al., 2019):

(1) The number of free parameters to be estimated must be smaller than or equal to the number of observations, that is, the model is overidentified (positive degrees of freedom). Otherwise, the model does not have enough information for it to be estimated, so it becomes either underidentified (negative degrees of freedom) or justidentified (zero degrees of freedom). For example: the model in Figure 2 has 13 parameters to be estimated (six variances of measurement errors of items, six factorial weights, one covariance between factors) and six observable variables (items). Therefore, we have 21 observations (obs = 6[6 + 1]/2) and eight degrees of freedom (df = 21–13), that is, the model is over-identified (it has enough information to be estimated);
(2) All latent variables (factors and item measurement errors) must be associated with a scale. Item measurement errors are associated with a scale through the ULI constraint (*unit loading identification*) that sets the direct effect of the measurement error on the corresponding item to unit (1.0). In the case of factors, we have two options: (a) also impose the ULI constraint (fixing to the unit one of the direct effects of the factor on one of the items, usually the first item) or (b) impose a UVI constraint (unit variance identification), which sets the variance of the factors to the unit (1.0). Both methods produce the same results, but the second one has the advantage of simplicity (Kline, 2016). However, in some software (e.g., AMOS) the first method is adopted by default.

So that there is no problems in identifying the model, it is necessary to consider the following (Blunch, 2013; Kline, 2016; Hair et al., 2019): (a) each item is a measurement indicator of a single factor and not can be associated with any other; (b) each item has an associated error that must be independent, that is, there must be no correlations between the items’ errors; and (c) each factor must consist of a minimum of three items. According to Blunch (2013), correlations between errors can be admitted if this does not makes question the identification of the model. However, this practice (which is in fact very common among researchers to adjust models), indicates that the two items measure something in common that is not explicit in the model. Therefore, if there is no correlation between the errors, it is guaranteed that the items are independent (Kline, 2016).

Regarding the estimation method, the one that is most common in CFA is the maximum likelihood *(ML).* The main objective is to find the parameter estimates as if they were the real population, maximizing the likelihood of the covariance matrix of the data with the covariance matrix constrained by the model. In practical terms, through the chi-square test *(Chi-Square:*χ^2^), discrepancies between the two matrices are analyzed (Chou and Bentler, 1995; Byrne, 2016). However, the theory underlying the ML method (which uses a *t*-test to test a null hypothesis) assumes that the data have a multivariate normal distribution (Kahn, 2006; Kline, 2016). However, as this almost never happens with data from the field of psychology, this method may be inappropriate, so corrective measures must be taken (Chou and Bentler, 1995).

Hence, according to several authors (Hoyle and Panter, 1995; Byrne, 2016), it is recommended that researchers consult, present and interpret not only information on univariate distribution of data (*skewness*: asymmetry; *kurtosis*: flattening), but also information on the multivariate distribution (*Mardia’s coefficient: multivariate kurtosis*) (see: Mardia, 1970). If the normalized Mardia coefficient is greater than 5.0, it indicates that the data do not have multivariate normal distribution (Byrne, 2016), the most common corrective measure being the use of the χ^2^ robust statistical test (Chou and Bentler, 1995; Hu and Bentler, 1999; Bentler, 2007; Byrne, 2016), the so-called Satorra-Bentler SCALED χ^2^ (S-Bχ^2^) (see: Satorra and Bentler, 2001), and the robust standard errors, both corrected for the non-normality of the distribution of the data. These corrections should produce more satisfactory results (Chou and Bentler, 1995). Unfortunately this option is only available in a few (e.g., EQS). For that reason some authors suggest the use of another option (Byrne, 2016; Kline, 2016): bootstrapping – is a computer-based method of resampling that combines the cases in a data set in different ways to estimate statistical precision. According to Byrne (2016), the key idea underlying the Bollen–Stine bootstrap technique (see Bollen and Stine, 1992), is that it enables the researcher to create multiple subsamples from an original data base. The importance of this action is that one can then examine parameter distributions relative to each of these spawned samples.

Although the χ^2^ test (corrected or not) is the most traditional for the evaluation of models, since it was the first adjustment index to be developed (see: Joreskog and Sorbom, 1982), it is rarely used in applied research as the only one (Brown, 2015). In fact, over time, some of its weaknesses have been pointed out (Chou and Bentler, 1995; Hoyle, 1995; Kahn, 2006; Worthington and Whittaker, 2006; Bentler, 2007; Brown, 2015; Byrne, 2016; Kline, 2016; Hair et al., 2019), such as: (1) in many situations (e.g., sample too lange or too small, model complexity, non-normal distribution of data) statistical significance is compromised; (2) the test values are inflated with the sample size and models are rejected, even when the differences between the matrices are very small, which can lead to the rejection of good models; and (3) its value is very restricted to not rejecting the null hypothesis, that is, a statistically significant χ^2^ test supports the alternative hypothesis that there are differences between the matrices (the model does not fit the data well). In short, in addition to the issues of sample size sensitivity and model complexity, we are faced with a test that checks whether the model fits the data perfectly or not. Some authors call it an “*exact fit*” test (Barrett, 2007, p. 816), but it is a well known fact that the hypothesis that the model must exactly match the data will never be precise (Bentler, 2007).

For these reasons, evaluating a model based only on the χ^2^ test may not be the best approach, as there are other indices that provide varied information and are very useful in determining the fit of the model to the data. It is common to use and report the values of the χ^2^ test, other indices are usually used with more confidence (Brown, 2015), being the most defensive strategy for the evaluation of the models the consultation of the adjustment indices of multiple classes (Hoyle, 1995). In other words, it is the complementary use of the so-called “*approximation*” fit indices, which adjust the χ^2^ test statistics to the sample size and model complexity, indicating the degree of discrepancy with the data (Barrett, 2007, p. 819).

The most used approximation indices are (Hoyle and Panter, 1995; Worthington and Whittaker, 2006; Brown, 2015): (a) the absolute indices (*absolute fit:* refers to the degree to which the covariances implied in the model match the observed covariances through which the free parameters are estimated); and (b) incremental indices (*incremental fit:* refers to the degree to which the model in question is superior to an alternative model, usually a model where no covariances between variables are specified – null or independent model). According to Hoyle and Panter (1995, p. 165), absolute indices are typically measures of *“lack of fit,”* since an optimal fit is indicated by values close to zero, and incremental indices are *“goodness-of-fit”* measures, since values close to one indicate a great improvement of the model in relation to the alternative model.

Although this issue does not meet consensus in the literature (it can be seen that researchers use a multiplicity of indices to evaluate the models), there seems to be a sustained trend (with which the authors of the present article agree) for the use of the following adjustment indices (Kahn, 2006; Worthington and Whittaker, 2006; Bentler, 2007; Brown, 2015; Byrne, 2016; Kline, 2016; Hair et al., 2019):

1). Absolutes Fit

- *Chi-Square Test* (χ^2^). It must be accompanied by the degrees of freedom (df) and the level of significance (p). It assesses whether there are discrepancies between the data covariance matrix and the model covariance matrix. Non-significant *p* values (*p* > 0.05) indicate good fit. If the data do not have a normal distribution, corrective measures must be taken (e.g., Satorra-Bentler: S-Bχ^2^);
- *Normalized Chi-Square* (χ^2^/df). Corresponds to the chi-square value divided by the degrees of freedom. Reduces test sensitivity to sample size and model complexity. Values of χ^2^/df < 3.0 indicate reasonable adjustment (Arbuckle, 2013; Hair et al., 2019), although there is neither any consensus regarding this value, nor even regarding its use as an adjustment index (Biddle et al., 2001). In any case, we can also take into account that values lower than 2.0 indicate a good fit (Blunch, 2013). A value of 5.0 is the minimum acceptable (Bentler, 2002);
- *Standardized Root Mean Square Residual (SRMR)*. It represents the value of the residual mean that derives from the adjustment values between the correlation matrices (from the model and from the one observed in the data). Values of SRMR ≤ 0.08 indicate good fit (Hu and Bentler, 1999), but values up to 0.10 may be considered acceptable (Worthington and Whittaker, 2006; Kline, 2016);
- *Root Mean Square Error of Approximation (RMSEA)*. It must be accompanied by the 90% confidence interval (RMSEA 90% CI) that indicates its accuracy. This index, which compensates for the effect of complexity (sensitive to the number of parameters and insensitive to the sample size), expresses the degree of the “error” of the model, thus evaluating the extent to which it fits (or not) the data (Brown, 2015). Through the analysis of the discrepancies between the matrices, it indicates which is the approximation to the perfect model (Byrne, 2016). Values of RMSEA ≤ 0.06 indicate an adequacy of the model (Hu and Bentler, 1999), but normally the most used cutoff values are (Brown, 2015; Byrne, 2016; Kline, 2016): ≤0.05 good fit; ≤0.08 acceptable fit, ≤0.10 indicate a mediocre fit and >0.10 a poor (unacceptable) fit;

2). Incrementals Fit

- *Comparative Fit Index (CFI)*. It is derived from the comparison of the covariations of the hypothetical model with a base model (null or independent), that is, this index estimates the improvement of the specified model fit over a null model in which the variables are not correlated (Kahn, 2006). In addition, this index also contemplates the sample size. Values of CFI ≥ 0.95 indicate a good fit (Hu and Bentler, 1999). However, several authors (Marsh et al., 2004; Worthington and Whittaker, 2006; Brown, 2015; Kline, 2016) point to values equal to or greater than 0.90 as an acceptable adjustment value. In general, 0.95 somehow became the magic number indicating good-fitting models, even though no empirical evidence supported such a development (Hair et al., 2019).
- *Non-normed Fit Index (NNFI)*. Also known as the *Tucker-Lewis Index* (TLI), it is very similar to the CFI, so some of the above-mentioned authors recommend using only one of them (e.g., Hair et al., 2019). However, the NNFI does take the degrees of freedom into account, which includes a penalty function for free parameters that do not improve the fit (Brown, 2015; Byrne, 2016). Although conceptually similar, these indices make different corrections depending on sample size (CFI) and model complexity (NNFI) (Kahn, 2006). Furthermore, the CFI is a type 2 incremental index (the values assume a central distribution) and the NNFI is a type 3 (the values assume a non-central distribution) (Chou and Bentler, 1995), being recommended to use an index of each type (Hoyle and Panter, 1995). Values of NNFI ≥ 0.95 indicate a good fit (Hu and Bentler, 1999), but values equal to or greater than 0.90 are acceptable;

The cutoff values that were recommended as indicators of good fit, are those proposed by Hu and Bentler (1999), and emerged as a result of their model evaluation simulations as a function of several factors (e.g., sample size and model complexity). However, despite several authors accepting and recommending its use (Kahn, 2006; Markland, 2007; Brown, 2015), this issue is far from unanimous (Hair et al., 2019). In fact, Marsh et al. (2004) strongly encourage researchers, reviewers, and editors not to generalize the Hu and Bentler cutoff values. In their opinion, there is no doubt that these cutoff values have very strong empirical support, however, they should not be interpreted as universal golden rules, as we may run the risk of rejecting good models. According to Marsh et al. (2004), the implicit assumption that the higher the better is wrong, and can lead to dubious practices on the part of researchers just to be able to increase the fit values of the models (e.g., correlate measurement errors). Therefore, researchers should keep in mind that the suggested cutoff values for the fit indexes are general guidelines and not necessarily definitive rules (Worthington and Whittaker, 2006).

In short, the golden rule is that there is no golden rule (Markland, 2007). In any case, for this author, we can even adopt a more conservative posture, taking some precaution in interpreting and reporting the data. However, it is still advisable for researchers to use the criteria proposed by Hu and Bentler (1999). They are more restrictive than the previous recommendations, and therefore less likely to lead to the acceptance of “*ill-fitting models*” (Markland, 2007, p. 857).

Some of the recommendations mentioned have been considered by several authors in Portugal, in the validation of questionnaires (using the CFA) in psychology applied to several physical activity domains: sport (e.g., Monteiro et al., 2017, 2019; Cid et al., 2019), exercise (e.g., Cid et al., 2018, 2020a,b; Rodrigues et al., 2019, 2021a) and physical education (e.g., Cid et al., 2016; Rodrigues et al., 2020).

Regarding the individual parameters estimated in the model (parameter estimates), it is advisable to report the following information as good practices (Brown, 2015; Byrne, 2016; Kline, 2016; Hair et al., 2019):

(1) *Factor weight (FL: factor loading).* Direct effect that latent variables (factors) have on observable indicators (items). They must be consistent with the theory underlying the instrument, be significant (*p* < 0.05) and values equal to or greater than 0.5. According to Hair et al. (2019), ideally this value should be 0.7, but in any case, we recommend as an alternative the values suggested by these authors depending on the sample size (already mentioned above);
(2) *Coefficient of determination (SMC: squared multiple correlations*). Although it is influenced by the factorial weight (since it corresponds to its squared value), the coefficient of determination corresponds to the amount of variance of the item that is explained by the factor. Thus, the higher its value, the greater the proportion of variance that is explained and, consequently, the smaller the proportion of unexplained variance (Kline, 2016). If we subtract the coefficient of determination from 1 (1-SMC), we will obtain the value of the unexplained item variance (Brown, 2015; Kline, 2016);
(3) *Measurement errors (EV: error variance)*. The variance of measurement errors represents the combined effect of all other sources of influence on observed values, in addition to the factor the item is supposed to measure (Kline, 2016). According to this author, measurement errors reflect two types of single variance: random measurement error (unreliable value) and measurement error associated with the evaluation instrument;
(4) *Standard errors (SE)*. The standard error of the estimated parameter (e.g., of the factor weights) represents the estimate of how much the sampling error may be influencing that parameter, which in a way represents an estimate of the stability of the model (Brown, 2015). There is no clear-cut criterion by which we can consider whether a standard error is problematic (Brown, 2015; Byrne, 2016; Hair et al., 2019), but low standard errors imply considerable accuracy in the parameter estimation, and excessively high indicate problems of inaccuracy of the estimated parameter and instability in the model (Brown, 2015).

The main purpose of the CFA is to provide answers about the fit of the model to the data, that is, whether the model is valid or not. However, this evaluation process can also provide additional information aiming at solving problems or improving the model (Hair et al., 2019). The indices indicate the goodness-of-fit of the model, but they do not disclose the reasons why the model does not fit the data. For this, there are two statistical methods that are frequently used in the search for potential problem areas (Chou and Bentler, 1995; Hoyle and Panter, 1995; Brown, 2015; Byrne, 2016; Hair et al., 2019):

(1) *Residual values (residuals)*. They represent the discrepancy between the data covariance matrix and the model constrained covariance matrix. High residual values (standardized residual matrix values) associated with a given pair of parameters are indicators of possible problems, which are contributing to the model’s misfit. Standardized residual values less than ± 2.58 do not suggest problems (Brown, 2015; Byrne, 2016; Hair et al., 2019), and values greater than ± 4.0 represent an unacceptable degree of error, so the most likely consequence is deletion of one of the parameters involved. In any case, the examples given by Chou and Bentler (1995) and Byrne (2016) seem to suggest that some attention should be paid to the higher residual values of the matrix;
(2) *Modification indices.* These indices (e.g., in the case of the EQS program: *Lagrange Multiplier Test and Wald Test*; in the case of the AMOS program: *Modification Indexes*), provide information about the improvement of the model as a function of freeing or setting parameters (e.g., indications about cross-loadings or correlations between measurement errors), indicating the decrease in the χ^2^ value that is expected if this happens. Some attention should be paid to elevated values and/or values that are associated with significance levels of *p* < 0.05. However, modifications to the model should never be made based on this information alone (Brown, 2015; Hair et al., 2019).

In short, researchers should always bear in mind that the re-specifications made always have an impact on the theory underlying the model (Hair et al., 2019). According to these authors, if the modifications are minor, the theoretical integrity of the model may not be affected (they accept as reasonable the elimination of two out of 15 variables). But if the modifications are larger, the investigator must be aware that this may affect the theoretical integrity of the model and, consequently, result in a new instrument that must be tested in a new sample.

## Multigroup Analysis

The objective of the multigroup analysis is to evaluate if the structure of the measurement model is equivalent (invariant) in different groups that have different characteristics (e.g., male vs. female or sport vs. exercise domain or Portuguese vs. Spanish culture).

To test measurement invariance between groups, the best model fit resulting from the factor structure analysis was initially examined in all groups separately. Then several levels of measurement invariance were measured according to Morin et al. (2016). There are essentially four levels of measurement invariance and each of these levels builds upon the previous level by introducing additional equality constraints on the model parameters to achieve stronger forms of invariance. As each set of new parameters is tested, the parameters know to be invariant from previous levels are constrained. Hence, the process of analyzing measurement invariance was essentially the testing of a series of increasingly restrictive hypotheses.

Thus establishing the following criteria for invariance of the models (Marsh, 1993; Cheung and Rensvold, 2002; Byrne, 2016): (1) factorial model analysis of each of the groups individually (the model should have a good fit in each group); and (2) multigroup analysis by restricting the model parameters, considering the following types of invariance: free parameters model (i.e., configural invariance), fixed factorial measurement model (i.e., measurement invariance), fixed factorial and covariance measurement model (i.e., scale-invariance), fixed factorial, covariance and error measurement model (i.e., residual invariance). According to Marsh (1993) measurement is considered as a minimum criterion for the variance of the model and the last criterion (residual invariance) is not indicative of a lack of invariance of the model, and some authors even consider that the analysis of this criterion is infrequent due to it being too restrictive (Byrne, 2016).

According to Cheung and Rensvold (2002), the difference in values between the unrestricted model (free parameters) and the restricted model (fixed parameters), should be ΔCFI ≤ 0.01. According to Byrne (2016), many researchers consider that model invariance evaluation based solely on the difference of the chi-squared (Δχ2) test is too restrictive. From this perspective, Cheung and Rensvold (2002), presented proof that it may be more reasonable to base decisions on CFI differences (ΔCFI).

However, model comparisons could be made according to several assumptions: (1) differences in CFI would be ≤0.010 for configural invariance (Marsh et al., 2010), supplemented by a change of ≤0.015 in RMSEA or a change of ≤0.030 in SRMR would indicate invariance; (2) for measurement invariance, scale-invariance, and residual invariance, a change of ≤0.010 in CFI, supplemented by a change of ≤0.015 in RMSEA or a change of ≤0.010 in SRMR would indicate acceptable criteria for invariance (Chen, 2008). Among the indexes presented for acceptable measurement invariance, CFI was chosen as the main criterion because RMSEA and SRMR tend to over reject an invariant model when sample size is small, particularly when using SRMR for testing loading or residual variance invariance (Chen, 2008).

Some of the recommendations mentioned have been considered by several authors in Portugal, when analysis of measurement model invariance as include in questionnaire validation in sport and exercise psychology (e.g., Cid et al., 2020b; Monteiro et al., 2020; Rodrigues et al., 2021b; Teixeira et al., 2021).

## Conclusion

Translating and validating measuring instruments in sport and exercise psychology is not an easy task. It requires an intricate effort, which is not limited to a simple translation with the aim of making it only a valid and reliable measure. All researchers should adopt a methodology that is rigorous and robust enough to allow the entire process to be carried out in a sustained manner, from the embryonic stage of translation to the validation phase of the construct of the measure of the psychological attribute. Only in this way is it possible to avoid the existence of fragile and inadequate assessment instruments, which could jeopardize all the research that could be carried out with their use.

Bearing in mind this concern, Vallerand (1989) proposed a methodology for the cross-cultural validation of psychological instruments, based on a systematic approach in stages, so that the path of the researcher is illuminated, in a process that can still be very subjective. With this methodology as a background, Fonseca and Brito (2005) promoted one of the first reflections published in Portugal on this issue applied to the sport context. In these authors’ opinion, in general, the procedures used by researchers did not seem uniform and should be criticized. Although in some cases complex validation processes were used, in others they did not pass beyond a simple translation, followed by the negative consequences as a result.

Although some of the Fonseca and Brito (2005) criticisms remain relevant today, since 2005 many advances have been made in questionnaires validation, and we have excellent examples of good practices as indicated before.

There is no doubt that SEM/CFA, which is a powerful statistical approach in evaluating models, has become increasingly popular, particularly in social and behavioral sciences (Biddle et al., 2001). According to these authors, this type of analysis provides a comprehensive set of tools that, when used sensibly, can substantially improve our understanding of models, promoting theoretical and applied development in sport and exercise psychology. However, researchers have to understand that the instrument validation process requires some investment, training and practice (Kahn, 2006), as there is still a large variety of practices, which is indicative of the need for even more rigor and standardization of procedures (Worthington and Whittaker, 2006). Many researchers use structural equation modeling without proper preparation for understanding that evaluating a model is a time-consuming process that is fraught with many kinds of difficulties and that, invariably, requires an enormous amount of work to achieve a good fit (Barrett, 2007). Therefore, it is essential that researchers devote considerable attention to models and that they choose the strategies to test them very carefully (Biddle et al., 2001).

In this sense, we hope that this reflection may have contributed in some way to help researchers in this complex task of translating and validating questionnaires. Whatever decisions be taken during this process, they will always be the responsibility of the researcher (Tabachnick and Fidell, 2019). For this reason, we should always bear in mind that the greatest benefit of making the right decisions is the possibility of increasing the chances of obtaining a set of clearer and more interpretable results. Yet, we must also not forget that the consequences of making less correct decisions usually lead to ambiguous and wrong results (Preacher and MacCallum, 2003).

## Author Contributions

LC, DM, FR, and DT were responsible for design and conceptualization. LC, DM, and FR wrote the first draft of the manuscript. DT and AE wrote the final draft of the manuscript. TB, AV, NC, and AA reviewed and critiqued the manuscript. All authors made relevant contributions and approved the final version of the manuscript.

## Conflict of Interest

The authors declare that the research was conducted in the absence of any commercial or financial relationships that could be construed as a potential conflict of interest.

## Publisher’s Note

All claims expressed in this article are solely those of the authors and do not necessarily represent those of their affiliated organizations, or those of the publisher, the editors and the reviewers. Any product that may be evaluated in this article, or claim that may be made by its manufacturer, is not guaranteed or endorsed by the publisher.
